# Commencement and Valedictory

**Published:** 1857-08

**Authors:** 


### COMMENCEMENT AND VALEDICTORY.

The Commencement Exercises in the Atlanta Medical College will take place on Thursday the 3d of September, upon which occasion the Valedictory Address to the graduating class will be delivered by Thomas S. Powell, M. D., of Sparta, Georgia.

